# How dietary arachidonic- and docosahexaenoic- acid rich oils differentially affect the murine hepatic transcriptome

**DOI:** 10.1186/1476-511X-5-10

**Published:** 2006-04-20

**Authors:** Alvin Berger, Matthew A Roberts, Bruce Hoff

**Affiliations:** 1 Department of Nutrition, University of North Carolina, Chapel Hill, NC 27599, USA; 2 Head of Biochemistry, Metabolon, Inc., 800 Capitola Drive, Suite 1, Durham, NC 27713, USA; 3 Director, Nestle Corporate Venture Funds, Acquisitions & Business Development, Nestle S.A., 55 Avenue Nestle, 1800 Vevey, Switzerland; 4 Director of Analytical Sciences, BioDiscovery, Inc., 100 North Sepulveda Blvd., Suite 1230, El Segundo, CA 90245, USA

## Abstract

**Introduction:**

Herein, we expand our previous work on the effects of long chain polyunsaturated fatty acids (LC-PUFA) on the murine hepatic transcriptome using novel statistical and bioinformatic approaches for evaluating microarray data. The analyses focuses on key differences in the transcriptomic response that will influence metabolism following consumption of FUNG (rich in 20:4n6), FISH (rich in 20:5n3, 22:5n3, and 22:6n3) and COMB, the combination of the two.

**Results:**

Using a variance-stabilized F-statistic, 371 probe sets (out of 13 K probe sets in the Affymetrix Mu11K chip set) were changed by dietary treatment (P < 0.001). Relative to other groups, COMB had unique affects on murine hepatic transcripts involved in cytoskeletal and carbohydrate metabolism; whereas FUNG affected amino acid metabolism via CTNB1 signaling. All three diets affected transcripts linked to apoptosis and cell proliferation, with evidence FISH may have increased apoptosis and decreased cell proliferation via various transcription factors, kinases, and phosphatases. The three diets affected lipid transport, lipoprotein metabolism, and bile acid metabolism through diverse pathways. Relative to other groups, FISH activated cyps that form hydroxylated fatty acids known to affect vascular tone and ion channel activity. FA synthesis and delta 9 desaturation were down regulated by COMB relative to other groups, implying that a FA mixture of 20:4n6, 20:5n3, and 22:6n3 is most effective at down regulating synthesis, via INS1, SREBP, PPAR alpha, and TNF signaling. Heme synthesis and the utilization of heme for hemoglobin production were likely affected by FUNG and FISH. Finally, relative to other groups, FISH increased numerous transcripts linked to combating oxidative such as peroxidases, an aldehyde dehydrogenase, and heat shock proteins, consistent with the major LC-PUFA in FISH (20:5n3, 22:5n3, 22:6n3) being more oxidizable than the major fatty acids in FUNG (20:4n6).

**Conclusion:**

Distinct transcriptomic, signaling cascades, and predicted affects on murine liver metabolism have been elucidated for 20:4n6-rich dietary oils, 22:6n3-rich oils, and a surprisingly distinct set of genes were affected by the combination of the two. Our results emphasize that the balance of dietary n6 and n3 LC-PUFA provided for infants and in nutritional and neutraceutical applications could have profoundly different affects on metabolism and cell signaling, beyond that previously recognized.

## Background

Microarrays and related technologies such as RT-PCR have accelerated our ability to understand the effects of long chain polyunsaturated fatty acids (LC-PUFA) and their derivatives on the transcriptome, implied metabolome, and lipid signaling cascades in various species and tissues [1,2].

Transcription factors indicated thus far include peroxisome proliferator activated receptors (PPARs), hepatic nuclear-4α (HNF-4α), nuclear factor κβ (NF-κβ), retinoid X receptor α (RXRα), sterol regulatory element binding protein-1c (SREBP-1c), and liver X receptors (LXR) [1,3]. Several studies have examined effects of LC-PUFA on the focused and global transcriptome [4], with some examining the effects of n6/n3 LC-PUFA ratios using precursors of 20:4n6 (such as 18:2n6), and precursors of 22:6n3 (such as 18:3n3) or 22:6n3 itself [5-9]. We are not aware of works comparing arachidonic acid (AA), eicosapentaenoic (EPA)/docosahexaenoic acids (DHA), and the combination of AA and EPA/DHA in liver and other tissues of mice nor other organisms.

In the present study and previous works, we fed mice diets enriched with fungal oil enriched in AA (FUNG), fish oil (FISH), or a combination of the two (COMB). In our first study, we examined the microarray transcriptional profile in liver and hippocampus, focusing on genes affecting lipid metabolism via known transcriptional signatures (PPARs, SREBPS, etc.), and provided supporting lipidomic data [1]. We documented and refined our statistical approaches used to select differentially regulated genes [10,11]. Thereafter, we focused in hepatic and hippocampal genes implicated in: behavior [12]; cancer etiology [13,14]; and obesity [15]. Lastly, we examined changes to all differentially regulated genes in liver [16] and hippocampus [17]. These works have been described in reviews [3,18-21]. What is still lacking is a more comprehensive evaluation of how n6 and n3 LC-PUFA differentially affect the murine hepatic transcriptome and how such events might translate to affects on metabolism [4]. To address this challenge, we re-evaluated our original microarray data [1] using new statistical approaches, pathway mapping, and updated literature.

## Results and discussion

### Comparison of diets on a genomic scale

**Figure 1:**
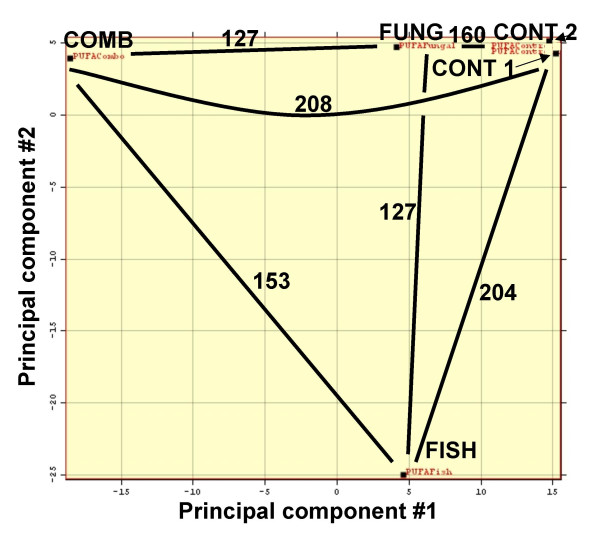
Principle component analysis scatter plot showing the five arrays used, represented by 371 most highly differentiated probe sets. Spatially closest arrays have the most similar genomic profiles. Super imposed are numbers of probes sets significantly different at P < 0.001 in six pair wise comparisons. Probes differing between arrays would not necessarily be the same in each comparison. PUFAConrol, Control diet; PUFAFungal, arachidonate-rich fungal oil; PUFAFish, fish oil; PUFACombo, combination diet.

In mouse liver, there were 371 probe sets varying between diets using an F-statistic (P < 0.001; GeneSight™ software (BioDiscovery, Inc.). Sets were evaluated by principal component analysis (PCA) analysis (Fig. 1) and retained 63% of the variance of the original data.

The number of significantly different genes between pairs of diets (F-statistic; P < 0.001) is superimposed on the PCA plot (Fig 1). The two replicate control (CONT) groups were very similar based on PCA, as expected. Numbers of pair wise probes differing between CONT and -FUNG, -FISH, and -COMB were 160, 204, and 208, respectively (P < 0.001; Fig. 1), indicating CONT and FUNG were most similar to one another. Numbers of probes differing between FUNG-FISH, FUNG-COMB, and FISH-COMB were 127, 127, and 153, respectively (P < 0.001). At P < 0.001, only 13 genes (0.001 × 13,000) are expected to appear by chance.

Using set intersection analysis on probes from pair wise comparisons, 20-, 27- and 44 probes differentiated FUNG, FISH and COMB from one another, respectively; most being down regulated (Table 1). 63349_s in FUNG-COMB, and C78039 in FUNG-FISH overlapped between sets.

**Table 1:** Number of up- and down-regulated probes that differentiated each treatment group from all other groups. Trends in regulation for probes differentiating treatment groups. The smallest set of probes (a subset of probes described in Fig. 1) differentiating each group (FUNG, FISH, COMB) from all others at P < 0.001 was determined with set intersection analysis. There is one row for each set of differentiating probes. Columns show numbers of genes in the list, up (+) and down (-) regulated. Almost all differentiating genes were down-regulated in pair wise comparisons.

		**Group vs.:**			
		
**GROUP**	**# Differentiating Genes**	**CONT**	**FUNG**	**FISH**	**COMB**
**FUNG**	20	+0/-20		+0/-20	+0/-20
**FISH**	27	+5/-22	+6/-21		+5/-22
**COMB**	44	+1/-43	+2/-42	+1/-43	

**Figure 2:**
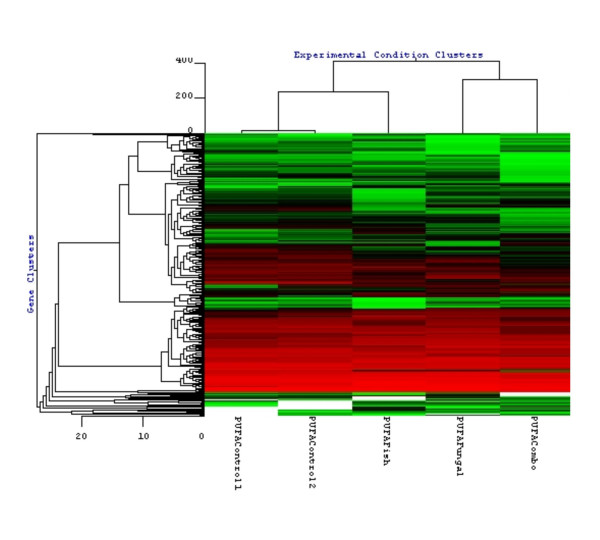
Hierarchical clustering of significant probes. Left, probe clusters; top, diet clusters; center, heat map, where red = high; black = middle; green = low. As expected, the two CONT groups clustered together.

Hierarchical cluster analysis on the 371 probes indicated the two CONT groups clustered together (Fig. 2). FISH was more similar to CONT than FUNG or COMB, which clustered together. However, cross bar height shows FISH is not profoundly closer to CONT, than FUNG or COMB.

**Figure 3:**
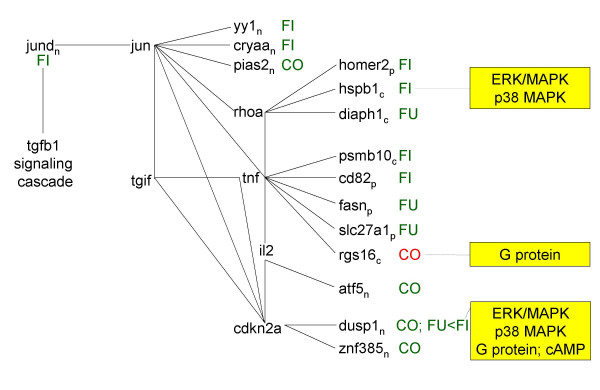
Pathway analysis. Figures 3–7 represent signaling pathways for 58 focus genes selected from Table 3 by Ingenuity Systems software (Redwood City, CA. The following signaling cascades are shown: JUN, TNF, and CDKN2A signaling cascade affecting: DNA replication; recombination and repair; immune response; and cell cycle (Fig. 3); TGFB1 signaling cascade affecting: cell morphology; cancer; and tumor morphology (Fig. 4); CTNB1 signaling cascade affecting: cell signaling; gene expression; and cell cycle (Fig. 5); INS1/hRAS signaling cascade affecting: carbohydrate metabolism; endocrine disorders; and metabolic disease (Fig. 6); and MYC signaling cascade affecting: viral function; gene expression; and cell Cycle (Fig. 7). Differentiating groups (per Table 3) are overlaid onto the signaling diagrams, and abbreviated: FU, fungal; FI, fish oil; CO, combination diet. When CO was the differentiating group, absolute differences between FU and FI are indicated. Intracellular location of focus genes (subscripts) are annotated: C, cytoplasm; E, extracellular; N, nucleus; P, plasma membrane; U, unknown. Major canonical functional/signaling categories associated with genes in the figures identified by the software, are shown in yellow boxes.

### Pathway analysis

**Figure 4:**
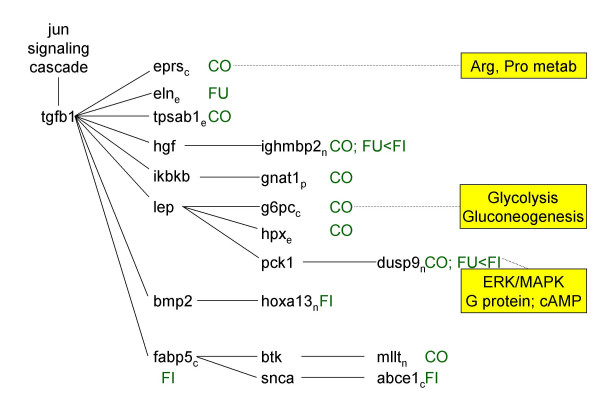
Pathway analysis. Figures 3-7 represent signaling pathways for 58 focus genes selected from Table 3 by Ingenuity Systems software (Redwood City, CA. The following signaling cascades are shown: JUN, TNF, and CDKN2A signaling cascade affecting: DNA replication; recombination and repair; immune response; and cell cycle (Fig. 3); TGFB1 signaling cascade affecting: cell morphology; cancer; and tumor morphology (Fig. 4); CTNB1 signaling cascade affecting: cell signaling; gene expression; and cell cycle (Fig. 5); INS1/hRAS signaling cascade affecting: carbohydrate metabolism; endocrine disorders; and metabolic disease (Fig. 6); and MYC signaling cascade affecting: viral function; gene expression; and cell Cycle (Fig. 7). Differentiating groups (per Table 3) are overlaid onto the signaling diagrams, and abbreviated: FU, fungal; FI, fish oil; CO, combination diet. When CO was the differentiating group, absolute differences between FU and FI are indicated. Intracellular location of focus genes (subscripts) are annotated: C, cytoplasm; E, extracellular; N, nucleus; P, plasma membrane; U, unknown. Major canonical functional/signaling categories associated with genes in the figures identified by the software, are shown in yellow boxes.

**Figure 5:**
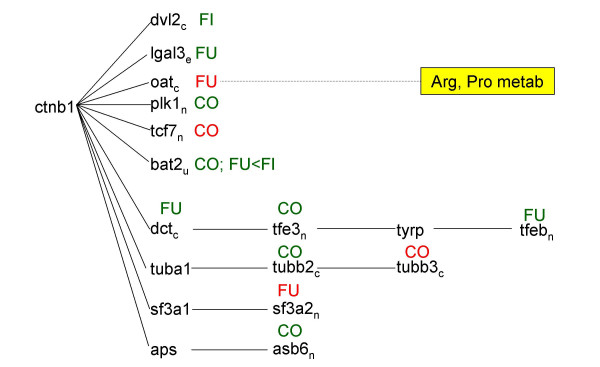
Pathway analysis. Figures 3-7 represent signaling pathways for 58 focus genes selected from Table 3 by Ingenuity Systems software (Redwood City, CA. The following signaling cascades are shown: JUN, TNF, and CDKN2A signaling cascade affecting: DNA replication; recombination and repair; immune response; and cell cycle (Fig. 3); TGFB1 signaling cascade affecting: cell morphology; cancer; and tumor morphology (Fig. 4); CTNB1 signaling cascade affecting: cell signaling; gene expression; and cell cycle (Fig. 5); INS1/hRAS signaling cascade affecting: carbohydrate metabolism; endocrine disorders; and metabolic disease (Fig. 6); and MYC signaling cascade affecting: viral function; gene expression; and cell Cycle (Fig. 7). Differentiating groups (per Table 3) are overlaid onto the signaling diagrams, and abbreviated: FU, fungal; FI, fish oil; CO, combination diet. When CO was the differentiating group, absolute differences between FU and FI are indicated. Intracellular location of focus genes (subscripts) are annotated: C, cytoplasm; E, extracellular; N, nucleus; P, plasma membrane; U, unknown. Major canonical functional/signaling categories associated with genes in the figures identified by the software, are shown in yellow boxes.

**Figure 6:**
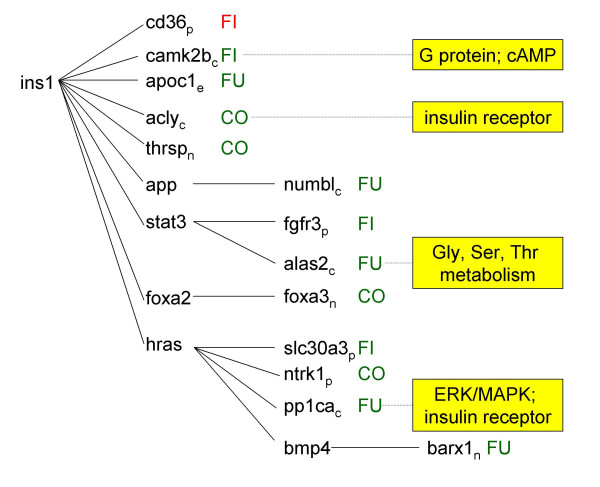
Pathway analysis. Figures 3-7 represent signaling pathways for 58 focus genes selected from Table 3 by Ingenuity Systems software (Redwood City, CA. The following signaling cascades are shown: JUN, TNF, and CDKN2A signaling cascade affecting: DNA replication; recombination and repair; immune response; and cell cycle (Fig. 3); TGFB1 signaling cascade affecting: cell morphology; cancer; and tumor morphology (Fig. 4); CTNB1 signaling cascade affecting: cell signaling; gene expression; and cell cycle (Fig. 5); INS1/hRAS signaling cascade affecting: carbohydrate metabolism; endocrine disorders; and metabolic disease (Fig. 6); and MYC signaling cascade affecting: viral function; gene expression; and cell Cycle (Fig. 7). Differentiating groups (per Table 3) are overlaid onto the signaling diagrams, and abbreviated: FU, fungal; FI, fish oil; CO, combination diet. When CO was the differentiating group, absolute differences between FU and FI are indicated. Intracellular location of focus genes (subscripts) are annotated: C, cytoplasm; E, extracellular; N, nucleus; P, plasma membrane; U, unknown. Major canonical functional/signaling categories associated with genes in the figures identified by the software, are shown in yellow boxes.

**Figure 7:**
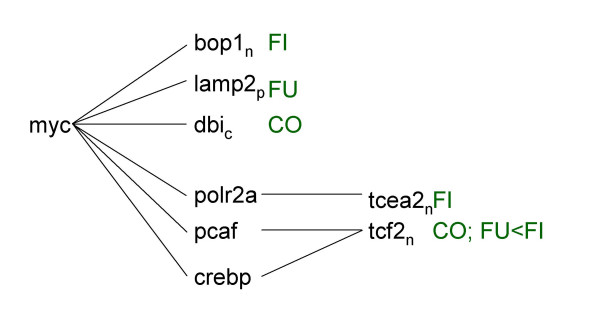
Pathway analysis. Figures 3-7 represent signaling pathways for 58 focus genes selected from Table 3 by Ingenuity Systems software (Redwood City, CA. The following signaling cascades are shown: JUN, TNF, and CDKN2A signaling cascade affecting: DNA replication; recombination and repair; immune response; and cell cycle (Fig. 3); TGFB1 signaling cascade affecting: cell morphology; cancer; and tumor morphology (Fig. 4); CTNB1 signaling cascade affecting: cell signaling; gene expression; and cell cycle (Fig. 5); INS1/hRAS signaling cascade affecting: carbohydrate metabolism; endocrine disorders; and metabolic disease (Fig. 6); and MYC signaling cascade affecting: viral function; gene expression; and cell Cycle (Fig. 7). Differentiating groups (per Table 3) are overlaid onto the signaling diagrams, and abbreviated: FU, fungal; FI, fish oil; CO, combination diet. When CO was the differentiating group, absolute differences between FU and FI are indicated. Intracellular location of focus genes (subscripts) are annotated: C, cytoplasm; E, extracellular; N, nucleus; P, plasma membrane; U, unknown. Major canonical functional/signaling categories associated with genes in the figures identified by the software, are shown in yellow boxes.

Pathway analysis (Figs. 3, 4, 5, 6, 7) was performed on selected transcripts differentiating the groups (Table 3; described in next section). It is not always possible from transcript data alone to correctly predict whether a pathway will be up or down regulated since post translational and dimerization events, and promoter/enhancer sequences ultimately affect DNA binding activation/signal transduction. The various diets provided different ratios of n6/n3 LC-PUFA. Thus, the dietary groups affected similar global signaling pathways; but differentially affected down stream signaling cascades and the magnitude/direction of change to specific transcripts. Generalized descriptions of the signaling cascades are provided below; detailed descriptions of specific transcripts are included in the subsequent section, "Comparison of diets on an individual gene level." Unreferenced literature emanates from Ingenuity software directly. Uppercase symbols refer to proteins; lower case symbols refer to genes; gene abbreviations are found in Table 3.

**Table 3:** Genes differentiating FUNG, FISH, AND COMB. Genes differentiating FUNG, FISH, and COMB. Shown are genes differentiating each group from all other groups, for which annotations were available. Unigene numbers lack Mm. prefix. Fold changes, relative to the differentiating group, are as follows: red, up regulation; green, down regulation. For example, for tcfe3, COMB was the differentiating group. Transcript levels for tcfe3 were 6.2 fold less for COMB relative to CONT; 7.8 fold less for COMB relative to FUNG; and 11.1 fold less for COMB relative to FISH; and FISH increased transcript levels relative to FUNG. Biological categories (e.g., Structural Role, Phosphatases and kinases) were derived from gene ontology (GO) nomenclature. Within each biological category, rows were sorted by differentiating group (FUNG, FISH, COMB), up or down regulation, and thereafter, alphabetically. ***P≤0.001; **0.001<P≤0.005; *0.005<P≤0.010. A more extensive version of this table including GO biological processes and GO molecular functions is available upon request.

				**Fold Change: Differentiating group vs. other groups:**			
				
**Gene Symbol**	**Name**	**Unigene**	**Differentiating Group**	**CONT**	**FUNG**	**FISH**	**COMB**
**Structural role (adhesion, microtubule, actin)**							

Sn	sialoadhesin	1374	COMB	-2.7***	-2.6**	-2.8***	
Catns	catenin src	198356	FUNG	-1.5*		-1.7**	-1.6*
Diaph1	diaphanous homolog 1 (Drosophila)	3282	FUNG	-3.5***		-4.9***	-4.2***
acta2	actin, alpha 2, smooth muscle, aorta	16537	COMB	-3.9***	-5.0***	-4.9***	
arhgef7	Rho guanine nucleotide exchange factor (GEF7)	3439	COMB	-3.4**	-3.7**	-3.8**	
cryaa	crystallin, alpha A	1228	FISH	-2.8***	-3.1**		-4.7***
homer2	homer homolog 2 (Drosophila)	228	FISH	-60.5***	-50.5***		-68.9***
tubb2	tubulin, beta 2	246377	COMB	-3.6***	-2.2**	-3.8***	
tubb3	tubulin, beta 3	40068	COMB	-2.7***	-2.0**	-2.9***	
eln	elastin	111845	FUNG	-19.0**		-16.6*	-19.1*
dvl2	dishevelled 2, dsh homolog (Drosophila)	5114	FISH	-29.4***	-47.3**		-21.9*
numbl	numb-like	49224	FUNG	-27.3***		-58.3***	-22.9***
hps1	Hermansky-Pudlak syndrome 1 homolog (human)	218381	COMB	-3.3***	-3.2**	-3.7***	
plod3	procollagen-lysine, 2-oxoglutarate 5-dioxygenase 3	137885	FUNG	-4.7***		-5.2***	-4.5***
hip1r	huntingtin interacting protein 1 related	149954	COMB	-9.1***	-8.2**	-7.0**	

**Phosphatases and kinases**							

ppp1ca	protein phosphatase 1, catalytic subunit, alpha isoform	1970	FUNG	-18.5***		-12.3***	-23.4***
ppp2cb	protein phosphatase 2a, catalytic subunit, beta isoform	7418	FISH	1.9***	1.6*		1.9***
ppm1b	protein phosphatase 1B, magnesium dependent, beta isoform	849	FISH	-27.4**	-48.3***		-57.5***
ptpn1	protein tyrosine phosphatase, non-receptor type 1	2668	FUNG	-10.3***		-28.4***	-13.3***
dusp1	dual specificity phosphatase 1	2404	COMB	-2.2**	-3.2***	-2.6***	
dusp9	dual specificity phosphatase 9	16479	COMB	-53.8***	-34.1***	-70.8***	
camk2b	calcium/calmodulin-dependent protein kinase II, beta	4857	FISH	-8.8***	-8.6***		-4.5**
ptpra	protein tyrosine phosphatase, receptor type, A	587	COMB	-10.2***	-12.2**	-8.0*	
fgfr3	fibroblast growth factor receptor 3	6904	FISH	-3.3**	-3.7**		-3.3*
plk1 (plk)	polo-like kinase homolog, (Drosophila)	16525	COMB	-1.9***	-2.1***	-2.0***	
ccnb1	cyclin B1	22569	FUNG	-46.7***		-19.5***	-58.6***
cdk4	cyclin-dependent kinase 4	6839	FISH	-2.8***	-2.5**		-3.4***
ntrk1	neurotrophic tyrosine kinase, receptor, type 1	129850	COMB	-2.84***	-3.31***	-3.7***	

**G protein coupled signaling**							

rgs16	regulator of G-protein signaling 16	181709	COMB	2.2***	1.9*	2.9***	
gnat1	guanine nucleotide binding protein, alpha transducing 1	69061	COMB	-20.4***	-18.8***	-17.9**	

**Transcription factors**							

notch2	Notch gene homolog 2, (Drosophila)	57005	FUNG	-5.0***		-6.6***	-3.4*
barx1	BarH-like homeobox 1	42241	FUNG	-23.5***		-62.5***	-15.1***
tcfeb (tfeb)	transcription factor EB	2305	FUNG	-4.3***		-6.5***	-5.1***
nfkbia	nuclear factor of kappa light chain gene enhancer in B-cells inhibitor, alpha	8884	FISH	-1.6**	-2.1***		-1.9***
jund1	Jun proto-oncogene related gene d1	1175	FISH	-7.2**	-18.6***		-14.0***
jun	Jun oncogene	482	FISH	-4.7***	-5.5***		-4.2***
yy1	YY1 transcription factor	3868	FISH	-3.2***	-5.7***		-4.4***
tef	thyrotroph embryonic factor	19258	FISH	-21.9***	-18.4**		-24.2**
usf2	upstream transcription factor 2	15781	FISH	-5.6***	-6.9***		-5.5***
hoxa13	homeo box A13	8056	FISH	-2.5***	-2.2*		-2.6**
tcea2	transcription elongation factor A (SII), 2	24245	FISH	-8.7**	-16.8***		-33.3***
foxa3; hnf-3g	forkhead box A3	42260	COMB	-2.2***	-2.8***	-2.4***	
tcf7	transcription factor 7, T-cell specific	31630	COMB	2.5**	2.1*	2.6**	
tcf2	transcription factor 2	7226	COMB	-55.0***	-46.5***	-89.1***	
atf5	activating transcription factor 5	1566	COMB	-3.1***	-1.9***	-2.0***	
gtf2i	general transcription factor II I	22593	COMB	-11.4***	-6.8**	-20.6***	
asb6	ankyrin repeat and SOCS box-containing protein 6	27656	COMB	-20.8***	-13.3**	-16.0***	
mllt1	myeloid/lymphoid or mixed lineage-leukemia translocation to 4 homolog (Drosophila)	148748	COMB	-9.8***	-15.0***	-12.2***	
ighmbp2	immunoglobulin mu binding protein 2	3179	COMB	-5.3***	-4.3*	-6.3***	
nr2c2	nuclear receptor subfamily 2, group C, member 2	3535	COMB	-37.9***	-81.7***	-19.72***	
tcfe3 (tfe3)	transcription factor E3	25762	COMB	-6.2***	-7.8***	-11.1***	
miz1 (pias2)	Msx-interacting-zinc finger	6370	COMB	-1.9*	-3.3***	-2.9***	
jmj	jumonji	25059	COMB	-8.7***	-14.6***	-7.1**	

**mRNA and rRNA splicing and processing**							

sf3a2	splicing factor 3a, subunit 2, 66kD	34039	FUNG	1.6**		2.0***	1.7**
nono	non-POU-domain-containing, octamer binding protein	21559	FUNG	-4.6***		-6.2***	-10.6***
srp9	signal recognition particle 9 kDa	89927	COMB	-3.1**	-2.7*	-3.3**	
bop1	block of proliferation 1	4283	FISH	-10.1*	-11.9*		-14.0*
zfp385 (znf385)	zinc finger protein 385	14099	COMB	-2.3***	-2.3***	-1.7**	

**Amino acid and protein metabolism**							

dct	dopachrome tautomerase	19987	FUNG	-4.8***		-4.5***	-2.1***
mrpl23	mitochondrial ribosomal protein L23	12144	COMB	-16.4***	-13.9***	-18.7***	
rpl28	ribosomal protein L28	3111	COMB	-1.8***	-1.5**	-1.8***	
eef2	eukaryotic translation elongation factor 2	27818	FISH	-4.9***	-3.0***		-4.0***
eef1a1 (2 probes)	eukaryotic translation elongation factor 1 alpha 1	196614	FUNG	-1.6*** to -1.7***		-1.6** to -2.1***	-1.5* to -1.9***
eprs	glutamyl-prolyl-tRNA synthetase	154511	COMB	-16.2***	-17.3***	-18.9***	
sars1	seryl-aminoacyl-tRNA synthetase 1	28688	FISH	-4.5***	-4.5**		-4.2*
ppil2	peptidylprolyl isomerase (cyclophilin)-like 2	218627	COMB	-3.4***	-4.6***	-3.7***	
ywhaq	tyrosine 3-monooxygenase/tryptophan 5-monooxygenase activation protein, theta polypeptide	14722	FUNG	-4.0**		-4.4*	-7.3***
oat	ornithine aminotransferase	13694	FUNG	1.7***		1.5*	1.7***
lamp2	lysosomal membrane glycoprotein 2	486	FUNG	-12.7***		-15.6***	-45.0***
ang	angiogenin (2 probes)	202665	FISH	-1.5* to -2.7***	-1.8*** to -4.5***		-1.8*** to -2.1*
kai1 (cd82)	kangai 1 (suppression of tumorigenicity 6, prostate)	4261	FISH	-1.7***	-2.3***		-1.5*
psmb10	proteasome (prosome, macropain) subunit, beta type 10	787	FISH	-22.7***	-47.8***		-14.0**
folh1	folate hydrolase	7522	FUNG	-10.8**		-13.3*	-21.1**
mcpt7 (tpsab1)	mast cell protease 7	3301	COMB	-3.5***	-2.9***	-3.5***	

**Lipid metabolism and P450s**							

acas2	acetyl-Coenzyme A synthetase 2 (ADP forming)	22719	COMB	-4.8***	-2.0*	-2.9***	
scd1 (2 probes)	stearoyl-Coenzyme A desaturase 1	140785	COMB	-1.5** to -1.8***	-1.4* to -1.7***	-1.6*** to -1.7***	
elovl3; cig30	elongation of very long chain fatty acids (FEN1/Elo2, SUR4/Elo3, yeast)-like 3	21806	FISH	1.9***	2.2***		3.4***
fasn	fatty acid synthase	3760	FUNG	-1.8***		-1.4*	-3.2***
acly (2 probes)	ATP citrate lyase (ACL)	25316	COMB	-3.6 to -5.2***	-1.9 to -2.0***	-2.0 to -2.3***	
lypla3	lysophospholipase 3	25492	FISH	-32.4***	-25.9***		-23.9***
thrsp	thyroid hormone responsive SPOT14 homolog (Rattus)	28585	COMB	-1.8***	-2.3***	-1.7***	
cyp4a10	cytochrome P450, 4a10	10742	FISH	4.7***	1.7**		1.7**
cyp24	cytochrome P450, 24	6575	FUNG	-8.6**		-15.1***	-19.3***

**Carbohydrate metabolism**							

lgals3	lectin, galactose binding, soluble 3	2970	FUNG	-4.0***		-5.5***	-4.8***
idua	iduronidase, alpha-L-	3054	COMB	-14.7***	-15.7***	-12.2**	
gaa	glucosidase, alpha, acid	4793	COMB	-3.1***	-2.5**	-2.3**	
g6pc	glucose-6-phosphatase, catalytic	18064	COMB	-1.9***	-2.3***	-3.0***	
pkm2	pyruvate kinase, muscle	2635	FISH	-23.3***	-48.8***		-12.2**
bat2	HLA-B associated transcript 2	20304	COMB	-9.6***	-4.8**	-7.7***	

**Transport (lipid and other)**							

abce1	ATP-binding cassette, sub-family E (OABP), member 1	5831	FISH	2.7***	2.5**		2.5**
fabp5	fatty acid binding protein 5, epidermal	741	FISH	-12.1***	-2.0**		-1.8***
hspg2	perlecan (heparan sulfate proteoglycan 2)	7257	COMB	-6.7***	-5.5***	-3.5**	
dbi	diazepam binding inhibitor	2785	COMB	-1.4**	-1.5**	-1.8***	
cd36	CD36 antigen	18628	FISH	2.4***	2.5***		3.8***
rab5c	RAB5C, member RAS oncogene family	29829	FISH	-3.6***	-3.6***		-4.0***
ap4s1	adaptor-related protein complex AP-4, sigma 1	116858	FUNG	-24.3*		-54.2***	-22.6*
slc4a2	solute carrier family 4 (anion exchanger), member 2	4580	FUNG	3.0**		3.9**	5.3***
slc10a1	solute carrier family 10 (sodium/bile acid cotransporter family), member 1	104295	FISH	-1.6**	-1.7**		-1.7**
apoc1	apolipoprotein C-I	182440	FUNG	-1.6***		-1.7***	-1.5**
apoe	apolipoprotein E	156335	FISH	1.5***	1.4*		1.8***
slc27a1	solute carrier family 27 (fatty acid transporter), member 1	7206	FUNG	-2.0**		-2.2**	-2.3**
apoa4	apolipoprotein A-IV	4533	COMB	-17.2***	-7.0***	-5.4***	
hba-a1	hemoglobin alpha, adult chain 1	196110	FISH	1.5***	1.4*		2.0***
hbb-b1	hemoglobin, beta adult major chain	233825	COMB	-1.7***	-1.7***	-2.3***	
aqp8	aquaporin 8	9970	COMB	-3.6***	-1.7**	-1.7**	

**Immune response/oxidative stress**							

c5r1	complement component 5, receptor 1	137488	FISH	-79.3***	-165.2***		-87.7***
cd72	CD72 antigen	88200	FISH	-46.2***	-38.9**		-41.1**
aldh1a7	aldehyde dehydrogenase family 1, subfamily A7	14609	FISH	1.9***	1.6**		1.6**
mif	macrophage migration inhibitory factor	2326	FISH	-2.3***	-3.1***		-1.9***
prdx2	peroxiredoxin 2	42948	FISH	-1.6***	-1.7**		-1.6**
hspb1	heat shock protein 1	13849	FISH	-4.0***	-5.8***		-6.3***
hspcb	heat shock protein 1, beta	2180	FUNG	-1.5*		-1.6*	-1.7**
hpxn	hemopexin	3485	COMB	-1.5***	-1.9***	-1.5**	

**Heme synthesis**							

alas1	aminolevulinic acid synthase 1	19143	FUNG	3.9***		2.1***	1.6**
alas2	aminolevulinic acid synthase 2, erythroid	140509	FUNG	-2.7**		-2.9**	-2.8**

**Calcium, sodium, zinc ion binding**							

tpt1	tumor protein, translationally-controlled 1	254	FUNG	-1.6**		-1.7**	-1.6*
fxyd2	FXYD domain-containing ion transport regulator 2	22742	COMB	-29.9***	-57.5***	-20.8*	
zfp312	zinc finger protein 312	34644	FISH	-6.0*	-8.2*		-12.8**
slc30a3	solute carrier family 30 (zinc transporter), member 3	1396	FISH	-4.2**	-5.3**		-6.8***
s100a3	S100 calcium binding protein A3	703	COMB	-3.0***	-4.0***	-4.4***	
rhbdl4	rhomboid like gene 4 (Drosophila)	219535	FISH	-3.3**	-4.3***		-3.4**

**Miscellaneous**							

serpinc1	serine (or cysteine) proteinase inhibitor, clade C (antithrombin), member 1	30025	FISH	1.7***	1.8***		2.5***
ssr2	signal sequence receptor, beta	7091	FUNG	-5.0***		-8.0***	-5.5**
ssr1	signal sequence receptor, alpha	138725	FISH	-6.2***	-5.9***		-6.3***
surf1	surfeit gene 1	6874	FISH	-2.4***	-2.4**		-2.3**
wbp1	WW domain binding protein 1	1109	FISH	-3.9***	-2.6***		-3.5***

### JUN/TGIF/TNF/CDKN2A signaling (Fig. 3)

JUN/TGIF/TNF/CDKN2A proteins affect DNA replication, recombination and repair, immune responses, and cell cycle. JUN is involved in signaling cascades including: B cell, chemokine, EGF, hypoxia, IGF-1, IL-6, neurotrophin/TRK, TGFβ, and toll-like signaling. Down stream of JUN, TGIF is involved in death receptor signaling; TNF has roles in apoptosis and cAMP signaling; CDKN2A is involved in G2/M DNA damage check point regulation.

FUNG and COMB increased jund relative to FISH (FISH deceased jund), similar to that reported in other models comparing effects of n6 and n3 LC-PUFA on jun [22]. Jund was decreased by FISH which in turn led to decreased yy1 and cryaa (crystallin, alpha A); decreased homer2 (homer homolog 2 Drosophila) and hspb1 (heat shock protein 1) (via RHOA); and decreased psmb10 (proteasome subunit, beta type 10) and cd82 (Kai1, kangai 1, suppression of tumorigenicity 6, prostate) (via TNF).

FUNG decreased diaph1 (diaphanous homolog 1) (via JUN target RHOA); decreased fasn (fatty acid synthase) and slc27a1 (solute carrier family 27 fatty acid transporter member 10) (via JUN target TNF) COMB decreased pias2 (miz1, Msx-interacting-zinc finger) (via JUN); increased rgs16 (regulator of G-protein signaling 16) (via JUN target TNF); and decreased dusp1 (dual specificity phosphatase 1) and znf385 (zinc finger protein 385) (via JUN target CDKN2A). Rgs16 is known to be decreased in response to G1/S activation in mouse liver, hence an increase could have a role in preventing cell progression [23].

### TGFB1 signaling (Fig. 4)

TGFB1 and JUN signaling are intertwined. TGFB1 is involved in IL-6 signaling and valine/leucine/isoleucine biosynthesis. FUNG decreased eln (elastin) (via TGFB1); and decreased ighmbp2 (immunoglobulin mu binding protein 2) relative to FISH (via TGFB1 target HGF). FISH decreased fabp5 (fatty acid binding protein 5, epidermal) (via TGFB1) and subsequently abce1 (ATP-binding cassette, sub-family E member 1) (via FABP-target SNCA); and decreased hoxa13 (homeo box A13) (via TGFB1 target BMP2). Human ABCE1 has recently been shown to be essential for in vitro and in vivo translation of mRNA, and to bind the initiation factors eIF2α and eIF5 [24].

COMB decreased ighmbp2, eprs (glutamyl-prolyl-tRNA synthetase), tpsab1 (mast cell protease 7) (via TGFB1), gnat1 (guanine nucleotide binding protein, alpha transducing 1) (via TGFB1 target IKBKb); and g6pc (glucose-6-phosphatase, catalytic) and hpx (hemopexin) (via TGFB1 target LEP).

### CTNB1 signaling (Fig. 5)

CTNB1 is involved in PI3K/AKT and WNT/β catenin signaling. FUNG decreased lgals3 (lectin, galactose binding, soluble 3), dct (dopachrome tautomerase), and bat2 (HLA-B associated transcript 2) (relative to FISH) and increased oat (ornithine aminotransferase) (all via CTNB1); it also increased sf3a2 (splicing factor 3a, subunit 2) (via CTNB1 target SF3A1). FISH decreased dvl2 (dishevelled 2, dsh homolog) (via CTNB1). COMB increased tcf7 (transcription factor 7, T-cell specific), and decreased plk1 (polo-like kinase homolog) and bat2 (all via CTNB1); and decreased tubb2 and increased tubb3 (via CTNB1 target TUBBA1).

### INS1/HRAS signaling (Fig. 6)

INS1 is involved in numerous signaling cascades: G protein, insulin, JNK/STAT, and PPAR. hRAS is an INS1 target involved in the following signaling cascades: B and T cell, EAF, estrogen, FGF, IL-2, insulin, integrin, neurotrophin/TRK, PI3K/AKT, SAPK/JNK, sterol biosynthesis, TGFβ, and VEGF. In response to growth factors, hRAS signaling involves the following activation cascade: hRAS-RAF(MAPKKK)-MEK (MAPKK)-ERK (MAPK).

FUNG decreased apoc1 (apolipoprotein C-I) (via INS1); decreased numbl (numb-like) (via INS1 target APP); decreased alas2 (aminolevulinic acid synthase 2, erythroid) (via INS1 target STAT3); and decreased pp1ca (protein phosphatase 1, catalytic subunit, alpha isoform) (via INS1 target hRAS).

FISH increased cd36 (CD36 antigen) and decreased camk2b (calcium/calmodulin-dependent protein kinase II, beta) (via INS1); decreased fgfr3 (fibroblast growth factor receptor 3) (via INS1 target STAT3); and decreased slc30a3 (solute carrier family 30) (via INS1 target hRAS). With respect to this last observation, DHA and fish oil can decrease RAS plasma membrane localization, RAS-GTP binding, and p42/44 ERK signaling in colonocytes [25].

COMB decreased acly (ATP citrate lyase) and thrsp (via INS1); decreased foxa3 (forkhead box A3) (via INS1 target FOXA2); and decreased ntrk1 (neurotrophic tyrosine kinase, receptor, type 1) (via INS1 target HRAS).

### MYC signaling (Fig. 7)

MYC is involved in various signaling cascades: cell cycle G1/S check point regulation, p38 MAPK, and PDGF. Down stream of MYC, CREBP is a PPAR target involved in TGFβ and NFKβ signaling.

FISH decreased bop1 (block of proliferation 1) (via MYC) and decreased tcea2 (via MYC target POL2RA). Consistent with our findings, fish oil can decrease cMYC protein [26]. FUNG decreased lamp2 (via MYC); and decreased tcf2 relative to FISH (transcription elongation factor A SII) (via MYC target PCAF). COMB decreased dbi (diazepam binding inhibitor) (via MYC) and decreased tcf2 (transcription factor 2) (via MYC targets PCAF and CREBP).

### Comparison of diets on an individual gene level

Herein, we focus on genes implicated in pathway analysis (Figs 3, 4, 5, 6, 7) and linkable to specific functions (Tables 2, 3). For brevity, signaling cascades cannot be described in their entirety. In Table 2, pathways are selected based on enrichment analysis. Table 3 includes genes that are individually most differentially regulated between diets. Up and down regulation are always relative to the other two groups even if not explicitly stated for brevity (e.g., down regulation by FUNG indicates down regulation by FUNG relative to FISH and COMB). Unreferenced literature emanates directly from Ingenuity- or Affymetrix Netaffx programs. For readability, gene and protein abbreviations are followed by full names in brackets.

**Table 2:** Genes differentiating FUNG, FISH, and COMB, using gene ontology classifications and enrichment analysis. Genes differentiating FUNG, FISH, and COMB, using gene ontology (GO) classifications and enrichment analysis. Differentially regulated genes (GEA model) were subjected to enrichment analysis to select GO terms "enriched with" these genes. Two broad GO categories included are "Biological Process" and "Molecular Function". Specific GO terms are included and specific genes within each gene term differentiating the diets are parenthesized, alphabetized, and separated by commas. Rows are sorted for consistency with Table 3 where possible. Differentiating genes were selected at P < 0.001, and GO terms computed at P < 0.01; P is the probability a random gene for each GO term will have as many genes with the same GO term as the actual list. The biological process category "Metabolism" was excluded as it was too general. Genes in Tables 2-3 are not identical because of the different statistical approaches utilized. Enrichment analysis placed acadm (acetyl-Coenzyme A dehydrogenase, medium chain (MCAD) in both the FA oxidation and Electron transport categories; it was removed from the latter.

	**Process/Differentiating group(s)**	**FUNG**	**FISH**	**COMB**
**Biological process classifications**	Amino acid metabolism	agxt, alas2, bcat1, oat		
	Acetyl-CoA biosynthesis	acas2, acly		acas2, acly
	Fatty acid and lipid synthesis			elovl2, elovl3, fasn, scd1
	Fatty acid oxidation	acadm, cpt1a, cpt2	acox1, cpt2, ech1	cpt1a, cpt2, acsl1(facl2)
	Carbohydrate metabolism			foxa3, gck
	Electron transport (Cyt P450 metabolism)	por, cyp2a4, cyp2b9, cyp3a11, cyp3a16, cyp3a41, cyp4a10		
	Lipid transport			apoa4, apoc2, osbpl5, pltp
	Heme biosynthesis		alad, alas1, alas2	
	Oxygen transport			hba-a1, hbb-b1
	Peroxidase reaction		gpx1, mpo, prdx2	
	Response to heat, stress, inflammation		hspa8, hspcb, hspb1, hspb8	

**Molecular function classifications**	Structural constituent of cytoskeleton			acta1, acta2, krt1-13, tuba1, tubb2, tubb3, tubb5
	Transaminase	agxt, bcat1, oat		
	Lyase	acly, fasn, pck1, umps		
	Transferase, transferring groups other than amino-acyl groups			elovl2, lce-pending
	Acyltransferase	alas1, alas2, cpt1a, cpt2, fasn, gpam		alas1, alas2, cpt1a, cpt2, fasn, gpam
	Monooxygenase/P450	cyp2a4, cyp2b9, cyp3a11, cyp3a41, cyp3a16, cyp4a10		
	5-aminolevulinate synthase	alas1, alas2	alas1, alas2	alas1, alas2
	Oxygen transporter			hba-a1, hbb-b1
	Oxidoreductase	acadm, aldh1a1, cyp2a4, cyp2b9, cyp3a11, cyp3a16, cyp3a41, cyp4a10, dct, fasn, gpx1, por, sdh1		
	Heat shock protein		cryac, hspa8, hspb1, hspcb	
	Acetate-coa ligase			acas2, facl2
	Aspartic-type endopeptidase	ctse, mela		
	RAS small monomeric gtpase	hras1, rras		
	Aldehyde dehydrogenase NAD^+^		aldh1a1, aldh1a7, aldh2	
	Antioxidant		prdx2, prdx4	
	Hormone		gh, igf2, prlpe, sct	
	Peptide hormone		igf2, npy, sct	
	Cathepsin S, L, K			ctsl, ctss
	Globin			hba-a1, hbb-b1
	Hydroxymethylglutaryl-CoA synthase			hmgcs1, hmgcs2

### Structural role

DIAPH1 has a role in actin cytoskeleton organization and biogenesis; and inhibits apoptosis [27,28]. FUNG down regulated diaph1 (diaphanous homolog 1) which may induce apoptosis.

FUNG down regulated plod3 (pro-collagen-lysine, 2-oxoglutarate 5-dioxygenase 3), coding lysyl hydroxylase, involved in collagen synthesis [29]. Fish oil consumption can increase collagen synthesis [30], and FISH could increase collagen synthesis via plod3. FUNG also down regulated eln (elastin) and catns (catenin src), having roles in cell adhesion.

FISH decreased homer2 involved in actin binding via glutamate receptor signaling. DVL2 (dishevelled 2, dsh homolog) acts on the cytoskeleton and induces apoptosis via MAPK9 and -10 and CTNB1/WNT/β/Catenin signaling (Fig. 5). FISH dramatically decreased dvl2 which may decrease apoptosis.

COMB differed from other groups with respect to effects on the cytoskeleton, using molecular function enrichment analysis (Table 2). Specifically, COMB down regulated: acta2 (actin, alpha 2) involved in smooth muscle contraction; arhgef7 (Rho guanine nucleotide exchange factor) and Sn (sialoadhesin) having roles in cell adhesion; and tubb2- and 3 (tubulins β 2 and -3), with roles in microtubule movement. Tubb 2 and 3 are known to be induced in response to G1/S activation in mouse liver [23].

### Phosphatases and kinases

PTP-1β (protein tyrosine phosphatase 1β, non-receptor type 1) is a tyrosine phosphatase which: interacts with EGF and PDGF receptors; regulates insulin and leptin signaling; and has affects on LDL-cholesterol and obesity in humans [31]. FUNG decreased ptpn1, which could affect cell proliferation.

CDK1 (cyclin dependent kinase 1) activation results in mitosis following CDK1-cyclin-B1 complex formation. FUNG decreased ccnb1 (cyclin B1) (proportionately increased by FISH), and this could impair CDK1 activation altering mitotic progression. CDK4 is a serine/threonine protein kinase required for G1-S cell cycle transition. Its activity is controlled by D-cyclins and CDK inhibitor p16 (INK4a). CDK4 also phosphorylates RB (retinoblastoma) protein which affects cell cycle progression. FISH down regulated cdk4 (cyclin-dependent kinase 4), which could inhibit cell cycle progression.

PPP1ca/PPP2C-β (protein phosphatases 1ca and -2b) are serine/threonine phosphatases with myosin phosphatase activity; and roles in glycogen metabolism, RNA processing, and cell cycle regulation. FISH increased ppp1ca and ppp2cb which could affect cell proliferation.

Camk2b (Calcium/calmodulin-dependent protein kinase IIβ, CaM-KII, CKII) is required for cell division via INS1 signaling (Fig. 6). Specifically, CKII phosphorylates SAG (sensitive to apoptosis gene), leading to degradation of IκBα and P27KIP1, and cell proliferation via G1/S phase transition [32]. SAG also inhibits lipid oxidant-induced apoptosis by inhibiting metal ion-induced release of cytochrome c and activating caspase. Camk2b was down regulated with FISH (and possibly its oxidation products acting on SAG), which may induce apoptosis/inhibit cell proliferation. CKII can also phosphorylate numb/numbl proteins in vitro; and in vivo, Cam-KI phosphorylates numbl in rat liver. Numbl was down regulated by FUNG (increased by FISH) via INS1-APP signaling (Fig. 6). The Cam-KII-NUMBL kinase cascade may affect axonal growth. NUMBL also interacts with additional binding proteins in liver (e.g., 14-3-3 proteins), thus new functions for numbl are being discovered [33]. CKII may also affect FA β-oxidation by altering CPT-1 (carnitine paltmitoyl transferase) phosphorylation [34].

DUSPs (dual specificity phosphatases or mitogen-activated protein kinase phosphatase, MKPs) negatively regulate MAPK/ERK-, SAPK/JNK-, and p38- mitogen activated protein kinase-induced cellular proliferation [35]. Dusps 1 and -9 were down regulated by COMB, which could increase proliferation [36].

PTPRA (protein tyrosine phosphatase, receptor type, A) de-phosphorylates and activates Src family tyrosine kinases, and may regulate integrin signaling, cell adhesion and proliferation. Ptpra was down regulated by COMB (increased with FISH) which could affect cell proliferation.

PLK1 (polo-like kinase homolog) acts through CTNB1 signaling (Fig. 5) to influence G2/M DNA damage check point regulation and induce mitotic entry of cells. NTRK1 (neurotrophic tyrosine kinase, receptor, type 1) acts through INS1/hRAS signaling (Fig. 6) to affect the cell cycle via protein tyrosine kinase signaling. Plk1 and ntrk1 were decreased by COMB. Plk is known to be increased in response to G1/S activation in mouse liver (induced by partial partial hepatectomy), hence a decrease in plk1could decrease cell progression [23].

### Transcription factors

Fish oil may decrease map kinase activity (p38, p44/42, JNK/SAPK) leading to decreased AP-1 (activator protein 1) binding activity, down regulated IL-6- and TNF signaling, and decreased mitosis [22,37,38]. jund1 was down regulated by FISH, which could inhibit cell cycle progression.

USF-2 (upstream stimulating factor 2) is an E-box binding factor which activates many of the same genes activated by the basic helix-loop helix (bHLH) transcription factor, SREBP-1. USF-2 is post-translationally phosphorylated [39] and may repress MYC-induced proliferation and transformation [40,41]. FISH down regulated usf2 (confirmed with real time-polymerase chain reaction, RT-PCR) [1], which could inhibit proliferation. USF-2 also binds fasn (fatty acid synthase) E-box [42]. Using a different gene selection model, FISH down regulated nfy (Nuclear factor Y). NF-Y binds Y-box motifs in FA synthase [1] (and inverted CCAAT/ATTGG motifs in peroxisomal β-oxidation transcripts) [43]. Taken together, FISH may down regulate fasn and decrease FA synthesis, via SREBP/SRE, USF-2/E-box, and NFY/Y-box interactions.

NFKBIA (nuclear factor of kappa light chain gene enhancer in B-cells inhibitor, alpha) inhibits apoptosis by inhibiting apoptosis activator NFKB1 (nuclear factor of kappa light chain gene enhancer in B-cells 1, p105). FISH down regulated nfkbia which could activate apoptosis.

HNF3γ is implicated in obesity, hyperlipidemia, and diabetes. It regulates glucagon transcription, insulin resistance, and pancreaticγ cell function [44]. Oxidative stress activates the related transcription factor FoxO3a, which in turn activates sterol carrier protein 2 (SCP2), protecting FA from further oxidation [45]; however, HNF3γ regulation is not known. COMB down regulated HNF3γ and the gluconeogenic transcript g6pc (glucose-6-phosphatase, catalytic (G6Pase), which could affect the balance between glycolysis and gluconeogenesis.

Activating transcription factors (ATF) are basic region-leucine zipper (bZIP) proteins [46]. ATF5 can repress human cAMP-induced gene transcription [47] and may inhibit apoptosis and promote G1/S transitions [23]. COMB decreased atf5 would could increase apoptosis and inhibit G1/S progression. Using a different gene selection model [1], COMB decreased the related factor, atf4 [48].

### Amino acid metabolism

Enrichment analysis indicated FUNG differed from other groups via changes to transcripts involved in amino acid metabolism and having transaminase function (Table 2).

Ornithine-delta-aminotransferase (OAT) has role in arginine/proline metabolism, and the urea cycle, acting through CTNB1 (Fig. 5). OAT converts L-ornithine to 2-ketoglutarate [49]. FUNG increased oat which could reduce ornithine for polyamine (putrescine, spermidine, and spermine) synthesis via ornithine decarboxylase, having subsequent affects on cell proliferation. DCT (dopachrome tautomerase) and SF3A1/SF3A2 (splicing factors 3a) also act through CTNB1 (Fig. 5) and affect tyrosine metabolism. FUNG decreased dct and increased sf3a 2.

EPRS (glutamyl-prolyl-tRNA synthetase) has roles in glutamate metabolism, and also death receptor signaling, cell cycle G1/S check point regulation, and leukotriene LTC_4 _synthesis. Eprs was down regulated by COMB via TGFB1 signaling (Fig. 4), which would affect amino acid metabolism and cell cycle regulation. TPSAB1 (mast cell protease 7) has roles in proteolysis and WNT/β catenin signaling. Tbsab1 was also down regulated by COMB via TGFB1 (Fig. 4).

### Fatty acid and phospholipid metabolism

As previously reported, LC-PUFA studied in our microarray experiments [1] likely increased FA β-oxidation via PPARα [50]; decreased FA synthesis via down regulation of SREBP and its signaling cofactors [51]; and increased glucose synthesis via PCK1 (phosphoenolpyruvate carboxykinase 1). In starvation experiments in mice, similar sets of transcripts were altered in the liver: apoa4, cyp4a14, and ech1 were increased; and scd1, fasn, and fabp5 were decreased [52].

In response to G1/S activation in mouse liver (induced by partial partial hepatectomy) [23], apoa4, cyp4a14, and ech1 were increased; and fasn, thrsp, and fabp5 were decreased [52]. LC-PUFA feeding thus mimicked the fasted metabolic state, and a state in which normally quiescent hepatocytes are dividing [53]. All groups up regulated mitochrondrial and peroxisomal FA β-oxidation transcripts (Tables 2, 3) [1], but no obvious trends concerning up or down regulation of β-oxidation emerged between groups in the present re-investigation.

Interestingly, COMB (providing a mixture of fatty acids in FUNG and FISH) differed most from the other groups with respect to transcripts involved in FA synthesis (Table 2). COMB down regulated: acetyl CoA biosynthesis via acas2 (acetyl-Coenzyme A synthetase 2); FA biosynthesis via thrsp (spot14); entry of AcCoA into the cytoplasm for FA synthesis via acly (ATP citrate lyase); and FA synthesis via fasn. Fasn was down regulated more with FUNG than FISH. COMB also down regulated scd1 (stearoyl-Coenzyme A desaturase 1) which could result in less monounsaturated FA being stored in triacylglycerol, cholesterol ester, and phospholipids (PL) pools, and potentially decreased obesity [54]. Our PL lipidomic data were consistent with the changes in SCD [1,15]. FISH increased FA elongation via up regulation of elovl3 (a long-chain fatty acyl elongase); this elongase may be SREBP1a-regulated [1,55].

LYPLA_2 _(lysophospholipase 3) hydrolyzes mitogenic lysoPL such as lysophosphatidylcholine [56]. FISH down regulated lypla2, which could increase PLA_2_-induced mitogenicity and affect other cellular processes.

### Cytochrome P450 metabolism

Using enrichment analysis (Table 2), FUNG affected numerous P450 transcripts, including por (P450 cytochrome oxidoreductase), cyp2a4, -2b9, -3a11, -3a16, -3a41, and -4a10 (up regulation of 4a10 confirmed with RTPCR; [1]).

CYP4 is activated by PPARα-RXR. Murine CYP4A-10 can form ω/ω-1 hydroxylated 20:4n6 and hydroxylated-epoxylated 20:4n6 [57,58]. Similar products from 20:5n3 and 22:6n3 have not been reported. These FA derivatives can affect: ion channel activity; regulate vascular tone and systemic blood pressures; and inhibit oxidative stress in mouse liver models [58,59]. FISH up regulated cyp4a10 (verified with RT-PCR) [1], and the related cyp 4a14 using a different gene selection model [1], which may result in the physiological changes noted above.

Calcitriol (1,25 (OH_2_) D_3_; vitamin D) binds vitamin D receptor (VDR). VDR-RXR heterodimers then bind VDRE (vitamin D-responsive elements) in genes in various organs including liver. Activated VDR regulates calcium and phosphate homeostasis and inhibits cell growth. In hepatocytes, via xenobiotic-responsive elements (ER6, DR3, and DR4), VDR also induces cyps 3a4, -2b6 and 2c9 [60].

Cyp24 degrades 1,25(OH)_2_D_3 _to 1,24,25(OH)_3_D_3_, a product with decreased hormonal activity. Cyp24 expression is activated by 1,25(OH)_2_D_3_, phorbol esters, and JNK (c-Jun N-terminal kinase), acting on VDRE [61]; and PXR (pregnane X receptor) agonists [62].

FUNG down regulated cyp24 which could increase 1,25(OH)_2_D, and decrease mitogenesis. FUNG did not up regulate cyps 2b6, -2c9, and -3a4, but up regulated family members, cyp2b9, -3a11 and -3a16, which are also major drug detoxifying enzymes [1].

### Lipid transport

FATP-1 (fatty acid transport protein 1; slc27a1) transports FA across the cell membrane in liver. Thereafter efflux is prevented by CoA esterification, via ACS (acyl CoA synthase) [63]. Slc27a1 and acs are PPARα regulated [63]. Slc27a1 was down regulated by FUNG, likely through PPARα, and this could lead to less FA transported into the liver for various purposes including triacylglycerol formation.

Through PPARα, CD36 is implicated in long chain free FA uptake in adipocytes and liver. CD36 also affects cell proliferation, angiogenesis, and tumor metastasis [64,65]; and is a receptor for thrombospondin, collagen type I, and oxidized LDL on macrophages [66]. Contrary to work in cancer cells where the fish oil component DHA reduced cd36 [67], herein, FISH up regulated cd36 which could increase uptake of FA into liver, and increase cell proliferation.

APOC1 inhibits lipoprotein lipase dependent triacylglycerol hydrolysis in mice, independent of very-low-density lipoprotein receptor and apoCIII [68]. FUNG decreased apoc1, which could lead to a lowering of circulating triacylglycerol.

E-FABP (fatty acid binding protein 5, epidermal) transports FA intracellularly in liver and other organs [69]. Fabp5 may be PPARα regulated based on clustering analysis [1] and the fact that a PPARα ligand decreased fabp [70]. Herein, FISH decreased fabp5 (verified with RT-PCR) [1], similarly to that reported in rats [70]. FISH-induced decrease in fabp5 could alter intracellular LC-PUFA transport [71].

Hepatic uptake of bile acids is mediated by various proteins including SLC (solute carrier family) members. Slc10a1 was down regulated by FISH, which could decrease hepatic uptake of bile acids during bile acid recycling, increasing circulating cholesterol levels [72]. Slc4a2 was up regulated by FUNG, which could affect biliary bicarbonate secretion, possibly via HNF signaling-HNF1α is regulated by Slc4a2 in human cells [73]. The related factor, hnf-3g, was increased by FUNG relative to COMB. AQP8 (aquaporin 8) facilitates hepatic bile secretion [74]. COMB down regulated aqp8 (aquaporin 8) possibly through SREBP1a [55,75]. Decreased AQP8 could decrease biliary cholesterol secretion and water secretion into bile [55] and conceivably increase circulating cholesterol.

COMB affected transcripts involved in apolipoprotein metabolism (apoa4, apoc2), PL transport (pltp, phospholipid transfer protein), and oxysterol binding (osbpl5, oxysterol binding protein-like 5) (Table 2, enrichment analysis). COMB down regulated apoa4 and apoa4 precursor (confirmed with RT-PCR) [1], likely through PPARα [70,76]. Changes to apoa4 mediated by COMB could affect reverse cholesterol transport from periphery to liver, since APOA4 is an HDL component.

APOE is involved in cholesterol transport and has anti-inflammatory properties [77]. The FISH-induced increase in apoe could enhance delivery of LDL cholesterol to liver and decrease serum triacylglycerol.

### Heme synthesis and oxygen transport

ALAS1 and -2 (aminolevulinic acid synthases 1 and -2) control the rate limiting steps in heme synthesis; ALAD (aminolevulinate, delta dehydratase) is the second enzyme in this pathway. Alas1 and alas2 were respectively increased- and decreased with FUNG, complicating the interpretation of how groups affected heme production.

There was evidence the diets affected heme utilization. Hbb-a1 (hemoglobin alpha, adult chain 1) and hbb-b1 (hemoglobin, beta adult major chain) are classes of hemoglobin. Heme is incorporated into not only hemoglobulins for oxygen transport; but also eicosanoid enzymes; and lipid peroxidases and heme-binding proteins which reduce damaging lipid peroxides [78]. Hbb-a1 was increased with FISH and hbb-b1 was decreased with COMB (FISH>FUNG) indicating more heme may be incorporated into hemoglobin with FISH relative to FUNG.

### Responses to oxidative stress and inflammation

FISH increased transcripts utilized to combat oxidative stress. This is expected since key FA in FISH having 5–6 double bonds vs. 2–4 double bonds for the key FA in FUNG, are more oxidizable [79]. Peroxidase transcripts increased by FISH included gpx1 (glutathione peroxidase 1), prdx2, prdx4 (peroxiredoxins), and mpo (myeloperoxidase). Peroxiredoxins are a new family of antioxidant proteins [80]. Prdx2 is a putative biomarker for progression of metastasis in melanoma cells, emphasizing its importance in controlling oxidative stress [81]. MPO is a heme-containing protein with roles in atherosclerosis and Alzheimer's disease [82].

Additional proteins with roles in oxidative stress were aldh1a7 (aldehyde dehydrogenases, increased with FISH), hspcb (a heat shock protein, decreased with FUNG), hspb1 (HSP25/P27, decreased with FISH), mif (macrophage migration inhibitory factor, decreased with FISH), c5r1 (complement component 5, receptor 1, dramatically decreased with FISH), cd72 (CD72 antigen, dramatically decreased with FISH), cryaa (crystallin, alpha A, decreased with FISH), and hpxn (hemopexin, decreased with COMB, FUNG>FISH) [78]. Using enrichment analysis (Table 2), additional transcription factors linked to oxidative stress distinguishing FISH, included: aldh1a1, aldh2 (aldehyde dehydrogenases), hspa8, and hspb8 (heat shock proteins). Globally, FISH had the most profound effects, increasing oxidative stress markers.

The increase in aldha7 was expected since more reactive aldehydes, such as 4-hydroxy-2-nonenal, are expected to be formed from reactions between oxygen radicals and the highly oxidizable LC-PUFA in fish oil, including DHA. Aldehyde dehydrogenases prevent lipid damage by destroying reactive aldehydes, and may be SREBP1a regulated [55,83]. Oxidant stress inducing drugs are known to increase aldh1a1 in rat liver, as well as transcripts for hsp (see below) [84]. Transcripts for aldehyde dehydrogenases, hsp, and cd36 (all were increased with FISH) are also known to increase with high fat feeding in mouse liver, consistent with the fact that high fat induces an oxidant stress [85].

Changes to HSPs would enable cell survival and recovery in response to oxidative stress and have been previously found to be changed following fish oil consumption [86]. HSPs also have roles in cytoskeletal actin dynamics in response to stress [87] and may account for changes to some cytoskeletal transcripts observed herein. Hspb1 encodes HSP25 and HSP27 [88]. LC-PUFA may affect transcription as well as post-translational phosphorylation of HSPs via ERK, PKCδ, TGFβ, p38, and MK2. MK2 (map kinase 2; also known as MAPKAPK2 or mitogen activated protein kinase-activated protein kinase 2) is activated by ERK. These signaling cascades are partly shown in Figs. 3–4. HSPs in turn activate transcription of antioxidant defense genes such as SOD2 (Mn superoxide dismutase). This in turn activates expression of inflammatory factors including TNFα(tumor necrosis factor α), IL1β (interleukin 1 β), and NFκβ (nuclear factor kappa beta) [89]. Changes to these transcripts up- and down stream of HSPs were not found but the upstream activator of HSPs, junD, was decreased with FISH (Fig. 3).

LC-PUFA, particularly from fish oil, affect neutrophil and macrophage functioning. Down regulation of mif (macrophage migration inhibitory factor) by FISH could increase hypersensitivity [90], opposite that expected for fish oil which typically down regulates immune responses. C5R1 (complement factor 5a receptor) promotes local inflammation and is a potent chemo-attractant for neutrophils and macrophages [91]. Its down regulation by FISH would decrease some inflammatory responses. CD72 prevents differentiation of naive B cells into plasma cells, blocking production of low-affinity antibodies. It also activates map kinase and BCR (B cell antigen receptor)-mediated calcium influx. Down regulation of cd72 with FISH may promote apoptosis/inhibit proliferation of B cells [92].

### Summary and conclusion (Table 4)

**Table 4:** Summary of genes differentiating FUNG, FISH, and COMB. Summary of genes differentiating FUNG, FISH, and COMB. Included are a subset of the most important genes selected from Table 3. The predicted global metabolic effect (up or down regulation) mediated by the differentiating group relative to other groups, is shown in the first column. The 2^nd ^column shows the differentiating group. The differentially regulated genes, and their direction of change, are shown in column 3, with signaling cascades in column 4. For example, relative to other groups, FUNG up regulated apoptosis via down regulation of the apoptosis inhibitor diap1, via JUN-RHOA signaling. TF, transcription factor.

**Metabolic effect of gene change**	**Differentiating group**	**Gene changes**	**Signaling pathway**
Amino acid: tyrosine & ornithine metabolism	FUNG	dct↓, sf3a2↑, oat↑	CTNB1
Protein synthesis	FUNG	eef1a1↓	

Apoptosis↑	FUNG	Diaph1↓ (apoptosis inhibitor)	JUN-RHOA
Apoptosis↓	FISH	dvl2↓	CTNB1/MAPK9 and -10 activation
Apoptosis↑	FISH	Camk2b↓	INS1/hRAS/SAG/*I*κBα, P27KIP1
Apoptosis↑	FISH	Nfkbia↓	NFκB signaling
Apoptosis↑/↓ G1/S progression	COMB	atf4↓, atf5↓	bZIP TF

Biliary bicarbonate secretion↓	FISH<FUNG	slc4a2↓	
Bile acid uptake by hepatocytes↓	FISH	slc10a1↓	
Biliary cholesterol secretion↓	COMB	aqp8↓	SREBP1a

Carbohydrate metabolism	COMB	foxa3↓, hnf3g↓, g6pc↓	foxa3: INS1-FOXA2
Carbohydrate metabolism	COMB; FUNG<FISH	bat2↓	CTNB1

Cell proliferation↑	FUNG<FISH	dusp9↓	JUN-CDKN2A. DUSP phosphatases inhibit MK
Cell proliferation↑	FUNG	ppp1ca↓	INS1-hRAS
Cell proliferation↑	FUNG>FISH	Ptpra↑	activates Src tyr kinases
Cell proliferation↑	FUNG<FISH	ptpn1↓, ppp2cb↓	
Cell proliferation↓	FUNG<FISH; COMB	ighmbp2↓	TGFβ 1-HGF
Cell proliferation↓	FUNG	cyp24↓ (↑1,25(OH)_2_D_3_)	VDRE-PXR signaling
Cell proliferation↓	FUNG	ccnb1↓ (codes cyclin B1)	CDK1 activation
Cell proliferation↓	FUNG	tcfeb↓, barx1↓, notch2↓	
Cell proliferation↓	FUNG<FISH; COMB	tcfe3↓	bHLH-zip TF
Cell proliferation↑	FISH	bop1↓	MYC
Cell proliferation↑/Lipid transport↑	FISH	cd36↑	PPARα signaling
Cell proliferation↑/Lipid transport↑	FISH	abce1↑	FABP-SNCA
Cell proliferation↓	FISH	tcea2↓	MYC-POL2RA
Cell proliferation↓	FISH	usf2↓	bHLH TF
Cell proliferation↓	FISH	jund↓, yy1↓	bHLHzip TF: IL6-TNF signaling
Cell proliferation↓	FISH	hoxa13↓	TGFβ 1-BMP2
Cell proliferation↓	FISH	fgfr3↓	INS1-STAT3
Cell proliferation↓	FISH	cdk4↓	D-cyclin-INK4a
Cell proliferation↓	FISH	tef↓	
Cell proliferation	FISH	rab5c↓ (phosphatase; GTPase)	
Cell proliferation	FISH	eef2↓ (activity depends on P state)	
Cell proliferation↓	COMB	plk1↓ (Zn finger)	CTNB1
Cell proliferation↓	COMB	Rgs16↑	JUN-TNF-G protein coupled receptor
Cell proliferation	COMB	atf5↓	bzip TF
Cell proliferation	COMB	ntrk1↓ (receptor tyr kinase)	INS1/hRAS
Cell proliferation	COMB	eprs↓ (activity depends on P state)	TGFβ 1-IKBKb

Clotting↓	FISH	serpinc1↑	
Collagen synthesis↓	FUNG	plod3↓	
Cytoskeletal effects	COMB	acta2↓, arhgef7↓, sn↓, tubb2↓, tubb3↓	CTNB1

FA β-Oxidation↑	ALL	acadm↑, crat↑, cpt1a↑, cpt2↑, ech1↑	PPARα signaling
AcetylCoA biosynthesis↓	COMB	acas2↓	
AcylCoa binding↓	COMB	dbi↓	MYC
FA synthesis/desaturation↓	COMB	acly↓ thrsp↓, fasn (FUNG<FISH)↓, scd↓	INS1, SREBP, PPARα, TNF
FA elongation↑	FISH	elovl2/3↑	

Cyt P450 metabolism↑	FISH	4a10↑, 4a14↑ (affect ion channel activity, vascular tone)	PPARα signaling
FA transported into liver↓	FUNG	slc27a1↓	JUN-TNF-PPARα
Lipid transport↓	FISH	fabp5↓	TGFβ 1-PPARα
Lipoprotein metabolism	FUNG	apoc1↓	INS1
Lipoprotein metabolism: reverse cholesterol transport↑	FISH	apoe↑ (↓ cholesterol and TAG)	
Lipoprotein metabolism: transport↓	COMB	apoa4↓	PPARα signaling

Galactose binding↓	FUNG	lgal3↓	CTNB1

Heme synthesis	FUNG	alas1↑, alas2↓	INS1-STAT3
Hemoglobin synthesis↑	FISH>FUNG	hba-a1↑, hbb-b1↑	

Immune system	COMB	tcf2↓, tcf7↑	MYC-PCAF-CREBP; CTNB1
Oxidative stress	COMB	hpx↓	TGFβ 1-LEP
Oxidative stress: aldehyde dehydrogenases↑	FISH	aldh1a7↑, aldh2↑ aldh1a1↑	SREBP1a
Oxidative stress: peroxidases↑	FISH	gpx1↑, prdx2↑, prdx4↑, mpo↑	
Oxidative stress: heat shock proteins	FISH vs FUNG	hspb1 (HSP25/27)↓, hspcb↑	ERK, PKCδ, TGFβ, p38, MK2/TNFα, IL1β, NFκB

#### Cytoskeletal-, amino acid-, heme- and carbohydrate metabolism

COMB decreased numerous transcripts implicated in the cytoskeleton, including acta2, arhgef7, sn, tubb2, and tubb3, via CTNB1 signaling. FUNG had the most profound affects on amino acid metabolism affecting tyrosine (dct, sf3a2) and ornithine (oat) metabolism, via CTNB1 signaling. Heme synthesis was likely altered via FUNG (alas1 increased, alas2 decreased; via INS1-STAT3 signaling). Hemoglobin synthesis may have been increased with FISH relative to FUNG (hba-a1, hbb-b1). Carbohydrate metabolism was most profoundly affected by COMB via decreases to foxa3 (INS1 signaling), bat2 (CTNB1 signaling), hnf3g, and g6pc.

#### Lipid metabolism and transport

All three diets increased numerous transcripts involved in FA β-oxidation (acadm, aldh1a1, aldh1a7, aldh2, crat, cpt1a, cpt2, and ech1) and activated by PPARα. FA synthesis (acly thrsp, fasn) and desaturation (scd) were down regulated by COMB relative to other groups, implying that a FA mixture of AA, EPA, and DHA is most effective at down regulating synthesis through INS1, SREBP, PPARα, and TNF signaling. FISH up regulated elongation of FA via elovl2/3. Lipid transport was also altered by the dietary groups. FUNG decreased slc27a1 which could decrease transport of FA into the liver; whereas FISH increased cd36 (via PPARα signaling), which could increase transport of FA into the liver. FISH decreased fabp5 which could affect intracellular fat transport (via TGFβ-PPARα signaling); and increased abce1 (via FABP-SNCA signaling). Understanding changes to apolipoprotein transcripts is complicated since mice and humans metabolize cholesterol and triacylglycerol differently (e.g., mice carry most of their cholesterol in HDL particles) and apolipoprotein polymorphisms have large impacts on cholesterol metabolism. FUNG decreased apoc1 (via INS1 signaling) which could lead to reduced circulating triacylglycerol. FISH increased apoe which could enhance delivery of cholesterol and triacylglycerol to liver, decreasing serum cholesterol and triacylglycerol. COMB decreased apoa4 (via PPARα signaling), which could affect transport of cholesterol to the liver. Bile acid metabolism was affected by all three diets. Relative to FUNG, FISH may have decreased biliary bicarbonate secretion (slc4a2) and bile acid uptake by hepatocytes (slc10a1); whereas, COMB may have decreased biliary cholesterol secretion (aqp8). Relative to other groups, FISH activated cyps 4a10 and -14 (via PPARα signaling), which hydroxylate AA and possibly DHA, forming products which affect vascular tone and ion channel activity.

#### Cell proliferation and apoptosis

Interpreting changes to transcripts implicated in cell proliferation is particularly complicated since transcribed proteins are ultimately regulated by pos-translational modifications and protein-protein interactions. FISH may have decreased cell proliferation via decreased tcea2 (MYC-POL2RA signaling), usf2 (bHLH transcription factor), junD, yy1 (bHLHzip transcription factor and IL6-TNF signaling), hoxa13 (TGFB1-BMP2 signaling), fgfr3 (INS1-STAT3 signaling), cdk4 (D-cyclin-INK4a signaling) and tef. FISH also decreased two phosphatase transcripts (rab5c, eef2) linked to cell proliferation; and altered three transcripts that might increase cell proliferation (decreased bop1, increased cd36 and abce1). FUNG may have increased cell proliferation via changes to phosphates including dusp9 (JUN-CDKN2A signaling), ppp1ca (INS1-hRAS), ptpn, and ppp2cb; and the kinase and ptpra (Src tyrosine kinase signaling). Contrastingly, FUNG could have also decreased cell proliferation via decreased ighmbp2 (TGFB1-HGF signaling), cyp24 (VDRE signaling), ccnb1 (less CDK1 activation), and tcfe3 (bHLH-zip TF). COMB also affected transcripts implicated in cell proliferation (see Table 4 for details). Relative to other groups, FISH likely increased apoptosis via camk2b (INS1/hRAS signaling), and nfkbia (NFκB signaling), but there was also evidence of changes to transcripts that would decrease apoptosis (e.g., via decreased dvl2). FUNG and COMB may also have increased apoptosis, but through different signaling cascades than noted for FISH.

#### Oxidative stress and inflammation

With respect to combating oxidative stress, relative to other groups, FISH increased various peroxidases (gpx1, prdx2, prdx4, mpo); increased an aldehyde dehydrogenase (aldh1a7, possibly via SREBP1a signaling); and affected heat shock proteins (hspb1 coding HSP25/27 was decreased, hspcb was increased). These observations are consistent with the major LC-PUFA in FISH (20:5n3, 22:5n3, 22:6n3) being more oxidizable than the major FA in FUNG (20:4n6; and small amounts of 22:4n6 and 22:5n6 formed endogenously following consumption).

#### Conclusion

Overall, diets rich in 20:4n6, 20:5n3/22:6n-3, and the combination of the two, had unique affects on the murine hepatic transcriptome, signaling cascades, and the predicted metabolome. The balance of dietary n6 and n3 LC-PUFA used in nutritional/neutraceutical applications could have profoundly different affects on metabolism and cell signaling, beyond that previously recognized. Future studies are needed to evaluate the effects of various ratios of n6 and n3 LC-PUFA on the transcriptome in diverse species, and in numerous tissues.

## Methods

Experimental diets, feeding and dissection conditions for obtaining mouse liver, nucleic acid preparation, and gene expression analysis using the Murine 11k GeneChip have been previously published [1,10,11]. The above information is summarized; new procedures are described in detail.

### Diets

Diets contained 90% fat-free AIN93G rodent diet, 0.4% milk fat, 1.2% palm olein, 1.9% sunflower oil, 1.5% soybean oil and 2.1-5.1% medium chain triacylglycerol oil [1]. Medium chain triacylglycerol oil in CONT was partly replaced with: 1.1% fungal oil (providing 0.5 dietary wt% AA and 1.0 en% AA) in FUNG; 1.9% fish oil (providing 0.5 dietary wt% DHA and 1.0 en% DHA) in FISH; and 1.1% fungal oil and 1.9% fish oil in COMB. AA and DHA levels were provided at levels known to affect neurotransmitter levels and behavior in rats [93]; and were not excessively high (2–3 fold higher than that recommended for infants, with slower Δ 6 desaturase activity than rodents).

### Statistical and gene selection procedures

#### Principle component analysis and hierarchical clustering

PCA was performed with GeneSight™ software (BioDiscovery, Inc.; Fig. 1; P < 0.001). Probe set measurements from Mu11k A and B arrays were combined, creating 13 K probe sets. To reduce data dimensionality, 371 probe sets differentiating the four groups from one another was pre-selected from the 13 K following: log transformation; centering values on each array by subtraction of the array mean value; and selecting probe sets whose expression was most significantly affected by diet, using an F-statistic. To address the paucity of replicate arrays (only two replicates for CONT), residuals derived from multiple genes were binned with a global error assessment (GEA) model [11]. Within-group variance was calculated per gene by combining 500 residual measurements from genes of similar intensity. Set intersection was used to find the smallest set of probes differentiating the four groups from one another (Table 1; P < 0.001). Tables 2, 3 described below include GEA-selected genes (P ≤ 0.001, 0.001 < P ≤ 0.005, 0.005 < P ≤ 0.010 as indicate by asterisks in the Table 3 legend). Hierarchical clustering included the 371 probes and used Euclidean distance metric and average linkage (Fig. 2).

#### Fold change calculations

Labeled RNA from 5 pooled mice per group was hybridized to Mu11K Affymetrix Chip arrays "A" and "B", each with 6.5 K probe sets. Expression values for each probe set were calculated with Affymetrix software. Data were log transformed discarding non-positive values, and log mean values subtracted to compare arrays. For CONT, two Mu11k chips were used to calculate a mean; a single Mu11k chip was used for other groups. Differential regulation for each dietary pair comparison was computed by subtracting normalized, log transformed values. Differential expression (DE) values were converted to fold change (FC) values: if DE > 0, FC = antilog(DE); if DE < 0, FC = -antilog(-DE).

### Enrichment analysis

Enrichment analysis was performed on differentiating gene lists, ranking gene ontology (GO) terms by frequency of occurrence (Table 2) [94], using BioDiscovery, Inc. software. Gene lists and GO term assignments from Affymetrix IDs were mapped to gene symbols to minimize false positives resulting when a large number of probe sets correspond to a single gene. Differentiating genes were selected at P < 0.001, and the most significant GO terms were computed at P < 0.01. P is the false positive rate, the probability of a random gene for each GO term having as many genes with the same GO term as the actual list [94].

### Consistency analysis

Chip sets A and B above had multiple oligonucleotide probe sets for some genes with Affymetrix's Unigene cluster mapping. Two differentially regulated genes (hba-a1, acly) had multiple probe sets but the direction of differential regulation was consistent in pair wise comparisons (Table 3).

### Pathway analysis (Figs. 3, 4, 5, 6, 7)

Pathway analysis was performed (Ingenuity Systems software, Redwood City, CA) to derive biological and signaling connections amongst the differentially regulated genes in Table 3. Of the 127 genes in Table 3, the program selected 66 focus genes having direct- and 132 having indirect connections, totaling 198 genes for pathway analysis. Direct connections require any two nodes (genes or gene products) to make direct physical contact. Indirect connections permit intermediate factors between nodes. The 198 genes were divided into 13 networks. Networks 1–5 had 15-, 12-, 13-, 13- and 5 focus genes, respectively. The remaining 8 networks had 1 gene each and were not considered. Network diagrams including these 58 focus genes and some connection genes were redrawn for simplicity (Figs. 3, 4, 5, 6, 7). The 5 networks were independent from one another, except for an indirect connection via JUN linking Figs. 3, 4. Abbreviations were from GeneCards [95].

### Functional, biological, and pathway information sources

Functional and biological information on genes was obtained from: Afffymetrix Netaffx Analysis Center [96]; EMBL Bioinformatic Harvester [97]; GeneCards [95,98]; MedMiner [99,100]; and NCBI Entrez Gene [101]. Additional pathway information was obtained from: Biocarta [102]; Cell Signaling Technology [103]; Genmapp [104]; Kegg [105]; MetaCyc ([106]; Reactome [107]; and RefViz [108].

## Abbreviations

AA, arachidonic acid; AKT, akt murine thymoma viral oncogene; AP-1, activator protein 1; APP, amyloid beta (A4) precursor protein; bHLH, basic helix-loop helix; BMP2, bone morphogenetic protein 2; bZIP, basic region-leucine zipper; CDK, cyclin dependent kinase; COMB, combination of FUNG+FISH; CONT, control; CPT-1, carnitine paltmitoyl transferase; CREBP, cAMP response element binding protein; CTNB1, catenin (cadherin-associated protein), beta 1; cyt, cytochrome; DE, differential expression; DHA, docosahexaenoic acid; EAF, ELL associated factor; EGF, epidermal growth factor; ERK, extracellular signal-regulated protein kinases; EPA, eicosapentaenoic acid; FA, fatty acid; FC, fold change; FGF, fibroblast growth factor; FISH, fish oil; FOXA2, forkhead box A2; FUNG, fungal; GEA, global error assessment; GO, gene ontology; HGF, hepatocyte growth factor; hRAS, Harvey rat sarcoma; IκBα inhibitor of kappa light polypeptide gene enhancer in B-cells; IKBKb, inhibitor of kappa light polypeptide gene enhancer in B-cells, kinase beta; IL, interleukin; INS1, insulin 1; JNK, c-Jun N-terminal kinase; JUN, jun proto-oncogene; LC-PUFA, long chain polyunsaturated fatty acids; LEP, leptin obesity homolog, mouse; LTC4, leukotriene C_4_; LXR, liver X receptor; LXRE, LXR responsive elements; MAPK, mitogen activated protein kinase; MK2, MAPKAPK2, MAPKAP kinase 2; MKP, mitogen-activated protein kinase phosphatase; MYC, myc myelocytomatosis viral oncogene; neurotrophin/TRK, neurotrophic tyrosine kinase, receptor; NF-κβ, nuclear factor κβ; P27, KIP1, cyclin-dependent kinase inhibitor 1B; p38, a MAPK; PCA, principal component analysis; PCAF, p300/CBP-associated factor; PDGF, platelet derived growth factor; PI3K, phosphatidylinositol 3-kinase; PKC, protein kinase C; PL, phospholipids; POL2RA, RNA polymerase II subunit; PPAR, peroxisome proliferator activated receptor; PXR, pregnane X receptor; RT-PCR, real time-polymerase chain reaction; RXR, retinoid X receptor; SAG (CKBBP1//ROC2/RBX2), sensitive to apoptosis gene; SAPK, stress-activated protein kinase (same as JNK); SF3A1, splicing factor 3a, subunit 1; SNCA, synuclein, alpha (non A4 component of amyloid precursor); SOD2, super oxide dismutase 2; SRE(BP), sterol regulatory element (binding protein); STAT3, signal transducer and activator of transcription 3; TF, transcription factor; TGFβ, transforming growth factor, beta; TGIF, transforming growth factor, beta, induced factor; TNF, tumor necrosis factor; VEGF, vascular endothelial growth factor; WNT, wingless-type MMTV integration site family. Gene symbol abbreviations for differentially regulated genes are found in Table 3 and the text.

## Competing interests

The author(s) declare that they have no competing interests.

## Authors' contributions

AB designed the animal experiments, provided overall coordination and wrote the manuscript; MR organized and conducted all laboratory and microarray analysis; MR with an extended team, developed the GEA gene selection model used in the present work; and BH provided all statistical and computational tools and performed the gene- and pathway- statistical analyses.
